# Supplementary material for: Association of HbA1c with VO2max in Individuals with Type 1 Diabetes: A Systematic Review and Meta-Analysis
Source: Metabolites. 2022 Oct 24;12(11):1017. doi: 10.3390/metabo12111017 (PMC9697838; doi:10.3390/metabo12111017)
Supplement: Supplementary file 1 [file metabolites-12-01017-s001.zip › metabolites-1948598-supplementary.pdf]

Supplemental Material

Univariate Meta-Regression

Univariate meta-regression for VO_{2max} vs. HbA_{1c} in relation to sex, age group and type of exercise only showed significant results for sex. The regression coefficient in studies including only women was -4.41 [-8.34 – -0.4] (p=0.028) while it was 7.71 [5.34 – 10.08] (p<0.001) in men. Regarding age groups, adolescents -0.85 [-7.08 – 5.39] (p=0.79) adolescents/adults 0.6 [-6.1 – 7.3] (p=0.86) and adults 0.16 [-5.82 – 6.14] (p=0.96). Once analyzed for exercise type cycling led to -3.49 [-7.48 -0.50] (p=0.08) and treadmill to 3.11 [-1.39 – 7.61] (p=0.18).

Table S1: Pubmed-search, july 2020

#	Term	Hit
#1	(diabetes mellitus, type 1 [mh])	75882
#2	(type 1 diabetes mellitus [tw])	10691
#3	(type 1 diabetes [tw])	87685
#4	("type i" diabetes mellitus [tw])	8695
#5	("type-i" diabetes mellitus [tw])	8695
#6	(T1DM [tw])	4613
#7	(IDDM [tw])	6869
#8	(insulin-dependent diabetes mellitus [tw])	15679
#9	(type 1 diabetic [tw])	5919
#10	(type i diabetic [tw])	1432
#11	(glycaemia [tw] OR glycemia [tw])	13387
#12	(diabet* AND (glycaemic control [tw])	8434
#13	(diabet* AND (glycemic control [tw])	22535
#14	(hba1c [tw])	38246
#15	(hemoglobin A1c [tw])	10042
#16	(glyc* hemoglobin [tw])	49847
#17	(eA1c [tw])	16
#18	#1 - #17 OR	173700
#19	(exercise test [tw])	68339
#20	(exercise [tw])	350086
#21	(cardiopulmonary exercise test [tw])	1646
#22	(incremental exercise test [tw])	850
#23	(cardiorespiratory exercise test [tw])	19
#24	(exercise [mh] AND (cardiopulmonary exercise test [tw] OR incremental exercise test [tw] OR cardiorespiratory exercise test [tw])	818
#25	(exercise AND (CPX [tw] OR IET [tw])	448
#26	(physical activity [tw] AND (cardiopulmonary exercise test [tw] OR incremental exercise test [tw] OR cardiorespiratory exercise test [tw])	167
#27	(graded exercise test [tw])	930
#28	(cardiopulmonary exercise testing [tw])	2965

#29	(incremental exercise testing [tw])	149
#30	(cardiorespiratory exercise testing [tw])	25
#31	(exercise testing [tw])	10721
#32	(physical fitness test [tw])	355
#33	(exercise performance test [tw])	22
#34	(bike test [tw])	32
#35	(bicycle test [tw])	177
#36	(cycling test [tw])	595
#37	(treadmill test [tw])	2770
#38	(running test [tw])	634
#39	(walking test [tw])	2792
#40	#19 - #39 OR	352395
#41	#18 AND #40	6805
#42	(exercise capacity [tw])	13060
#43	(maxi* o2 uptake [tw])	1007
#44	(maxi* 02 uptake [tw])	2
#45	(maxi* o2 consumption [tw])	948
#46	(maxi* 02 consumption [tw])	5
#47	(peak o2 consumption [tw])	128
#48	(peak 02 consumption [tw])	728
#49	(Physical capacity [tw])	1984
#50	(physical work* capacity [tw])	13043
#51	(Functional capacity [tw])	13301
#52	(maxi* aerobic capacity [tw])	2447
#53	(Vo2max [tw])	10097
#54	(v02max [tw])	40
#55	(Vo2peak [tw])	3325
#56	(v02peak [tw])	7
#57	(maxi* oxygen uptake [tw])	9502
#58	(maxi* oxygen consumption [tw])	21189
#59	(peak oxygen consumption [tw])	2941
#60	(lactate threshold [tw])	1441
#61	(aerobic threshold [tw])	97
#62	(anaerobic threshold [tw])	5235
#63	#42 - #62 OR	71614
#64	#18 AND #40 AND #63	708
#65	Limit to humans	621

Table S2: Embase-search, july 2020

#	Term	Hit
#1	(diabetes mellitus, type 1).mp.	3278
#2	(type 1 diabetes mellitus).mp.	16600
#3	(type 1 diabetes).mp.	64792
#4	("type i" diabetes mellitus).mp.	2507
#5	("type-i" diabetes mellitus).mp.	2507
#6	(T1DM).mp.	9363
#7	(IDDM).mp.	8084
#8	(insulin-dependent diabetes mellitus).mp.	346987
#9	(type 1 diabetic).mp.	8522
#10	(type i diabetic).mp.	1909
#11	(glycaemia OR glycemia).mp.	22066
#12	(diabet* AND glycaemic control).mp.	14360
#13	(diabet* AND glycemic control).mp.	58165
#14	(hba1c).mp.	73631
#15	(hemoglobin A1c).mp.	111441
#16	(glyc* hemoglobin).mp.	35241
#17	(eA1c).mp.	41
#18	#1 - #17 OR	440560
#19	(exercise).mp.	494013
#20	(exercise test).mp.	70214
#21	(cardiopulmonary exercise test).mp.	8003
#22	(incremental exercise test).mp.	1061
#23	(cardiorespiratory exercise test).mp.	31
#24	(exercise AND cardiopulmonary exercise test OR incremental exercise test OR cardiorespiratory exercise test).mp.	9033
#25	(exercise AND CPX OR IET).mp.	1475
#26	(physical activity AND cardiopulmonary exercise test OR incremental exercise test OR cardiorespiratory exercise test).mp.	1798
#27	(graded exercise test).mp.	1120
#28	(cardiopulmonary exercise testing).mp.	5883
#29	(incremental exercise testing).mp.	208
#30	(cardiorespiratory exercise testing).mp.	36
#31	(exercise testing).mp.	17027
#32	(physical fitness test).mp.	435
#33	(exercise performance test).mp.	30
#34	(bike test).mp.	45
#35	(bicycle test).mp.	297
#36	(cycling test).mp.	675
#37	(treadmill test).mp	5395
#38	(running test).mp.	729
#39	(walking test).mp.	5816
#40	#19 - #39 OR	499654
#41	#18 AND #40	21604
#42	(exercise capacity).mp.	21760
#43	(max* o2 uptake).mp.	760
#44	(max* 02 uptake).mp.	6
#45	(max* o2 consumption).mp.	572
#46	(max* 02 consumption).mp.	2
#47	(peak o2 consumption).mp.	222

#48	(peak 02 consumption).mp.	6
#49	(physical capacity).mp.	13122
#50	(physical work* capacity).mp.	1529
#51	(functional capacity).mp.	20882
#52	(max* aerobic capacity).mp.	1109
#53	(vo2max).mp.	10884
#54	(v02max).mp.	119
#55	(vo2peak).mp.	4815
#56	(v02peak).mp	49
#57	(max* oxygen uptake).mp.	7130
#58	(max* oxygen consumption).mp.	4841
#59	(peak oxygen consumption).mp.	4199
#60	(lactate threshold).mp.	1652
#61	(aerobic threshold).mp.	141
#62	(anaerobic threshold).mp.	5895
#63	#42 - #62 OR	82835
#64	#18 AND #40 AND #63	1744
#65	Limit to humans	1602
#66	Limit to english language	1546

Table S3: Web of Science-search, july 2020

#	Term	Hit
#1	TS= (diabetes mellitus, type 1)	74621
#2	TS= (type 1 diabetes mellitus)	74621
#3	TS= (type 1 diabetes)	142389
#4	TS= ("type i" diabetes mellitus)	6793
#5	TS= ("type-i" diabetes mellitus)	6793
#6	TS= (T1DM)	4652
#7	TS= (IDDM)	10663
#8	TS= (insulin-dependent diabetes mellitus)	17961
#9	TS= (type 1 diabetic)	54974
#10	TS= (type i diabetic)	14556
#11	TS= (glycaemia OR glycemia)	13760
#12	TS= (diabet* AND glycaemic control)	9620
#13	TS= (diabet* AND glycemic control)	40051
#14	TS= (hba1c)	24590
#15	TS= (hemoglobin A1c)	9757
#16	TS= (glyc* hemoglobin)	31070
#17	TS= (eA1c)	12
#18	#1 - #17 OR	231352
#19	TS= (exercise)	437491
#20	TS= (exercise test)	103364
#21	TS= (cardiopulmonary exercise test)	6531
#22	TS= (incremental exercise test)	6030
#23	TS= (cardiorespiratory exercise test)	4455
#24	TS= (exercise AND cardiopulmonary exercise test OR incremental exercise test OR cardiorespiratory exercise test)	15213
#25	TS= (exercise AND CPX OR IET)	1283
#26	TS= (physical activity AND cardiopulmonary exercise test OR incremental exercise test OR cardiorespiratory exercise test)	10372
#27	TS= (graded exercise test)	4873
#28	TS= (cardiopulmonary exercise testing)	6531
#29	TS= (incremental exercise testing)	6030
#30	TS= (cardiorespiratory exercise testing)	4455
#31	TS= (exercise testing)	103364
#32	TS= (physical fitness test)	13505
#33	TS= (exercise performance test)	27169
#34	TS= (bike test)	815
#35	TS= (bicycle test)	4215
#36	TS= (cycling test)	167889
#37	TS= (treadmill test)	14610
#38	TS= (running test)	77358
#39	TS= (walking test)	39691
#40	#19 - #39	692856
#41	#18 AND #40	10252
#42	TS= (exercise capacity)	46371
#43	TS= (max* o2 uptake)	752
#44	TS= (max* 02 uptake)	1830
#45	TS= (max* o2 consumption)	674
#46	TS= (max* 02 consumption)	1318
#47	TS= (peak o2 consumption)	257

#48	TS= (peak 02 consumption)	724
#49	TS= (physical capacity)	59911
#50	TS= (physical work* capacity)	10507
#51	TS= (functional capacity)	91334
#52	TS= (max* aerobic capacity)	6175
#53	TS= (vo2max)	5727
#54	TS= (v02max)	10
#55	TS= (vo2peak)	2101
#56	TS= (v02peak)	3
#57	TS= (max* oxygen uptake)	12801
#58	TS= (max* oxygen consumption)	12005
#59	TS= (peak oxygen consumption)	6483
#60	TS= (lactate threshold)	4745
#61	TS= (aerobic threshold)	3652
#62	TS= (anaerobic threshold)	5652
#63	#42 - #62 OR	199869
#64	#18 AND #40 AND #63	1481
#65	Limit to english language	1423

Table S4: Cochrane-search, july 2020

#1	(diabetes mellitus, type 1).mp.	5753
#2	(type 1 diabetes mellitus).mp.	1687
#3	(type 1 diabetes).mp.	6599
#4	("type i" diabetes mellitus).mp.	196
#5	("type-i" diabetes mellitus).mp.	196
#6	(T1DM).mp.	954
#7	(IDDM).mp.	589
#8	(insulin-dependent diabetes mellitus).mp.	20517
#9	(type 1 diabetic).mp.	758
#10	(type i diabetic).mp.	168
#11	(glycaemia OR glycemia).mp.	3305
#12	(diabet* AND glycaemic control).mp.	3583
#13	(diabet* AND glycemic control).mp.	10692
#14	(hba1c).mp.	17158
#15	(hemoglobin A1c).mp.	8149
#16	(glyc* hemoglobin).mp.	9318
#17	(eA1c).mp.	8
#18	#1 - #17 OR	44891
#19	(exercise).mp.	95428
#20	(exercise test).mp.	13359
#21	(cardiopulmonary exercise test).mp.	952
#22	(incremental exercise test).mp.	228
#23	(cardiorespiratory exercise test).mp.	13
#24	(exercise AND cardiopulmonary exercise test OR incremental exercise test OR cardiorespiratory exercise test).mp.	1188
#25	(exercise AND CPX OR IET).mp.	166
#26	(physical activity AND cardiopulmonary exercise test OR incremental exercise test OR cardiorespiratory exercise test).mp.	390
#27	(graded exercise test).mp.	254
#28	(cardiopulmonary exercise testing).mp.	771
#29	(incremental exercise testing).mp.	35
#30	(cardiorespiratory exercise testing).mp.	6
#31	(exercise testing).mp.	2288
#32	(physical fitness test).mp.	60
#33	(exercise performance test).mp.	16
#34	(bike test).mp.	13
#35	(bicycle test).mp.	68
#36	(cycling test).mp.	208
#37	(treadmill test).mp	962
#38	(running test).mp.	153
#39	(walking test).mp.	1801
#40	#19 - #39 OR	96465
#41	#18 AND #40	4751
#42	(exercise capacity).mp.	5418
#43	(max* o2 uptake).mp.	84
#44	(max* 02 uptake).mp.	0
#45	(max* o2 consumption).mp.	55
#46	(max* 02 consumption).mp.	0
#47	(peak o2 consumption).mp.	40
#48	(peak 02 consumption).mp.	0

#49	(physical capacity).mp.	1274
#50	(physical work* capacity).mp.	224
#51	(functional capacity).mp.	3943
#52	(max* aerobic capacity).mp.	241
#53	(vo2max).mp.	2618
#54	(v02max).mp.	18
#55	(vo2peak).mp.	1321
#56	(v02peak).mp	5
#57	(max* oxygen uptake).mp.	1658
#58	(max* oxygen consumption).mp.	1123
#59	(peak oxygen consumption).mp.	1018
#60	(lactate threshold).mp.	334
#61	(aerobic threshold).mp.	41
#62	(anaerobic threshold).mp.	1269
#63	#42 - #62 OR	17172
#64	#18 AND #40 AND #63	483
#65	Limit to english language	351

Figure S1: Funnel Plot

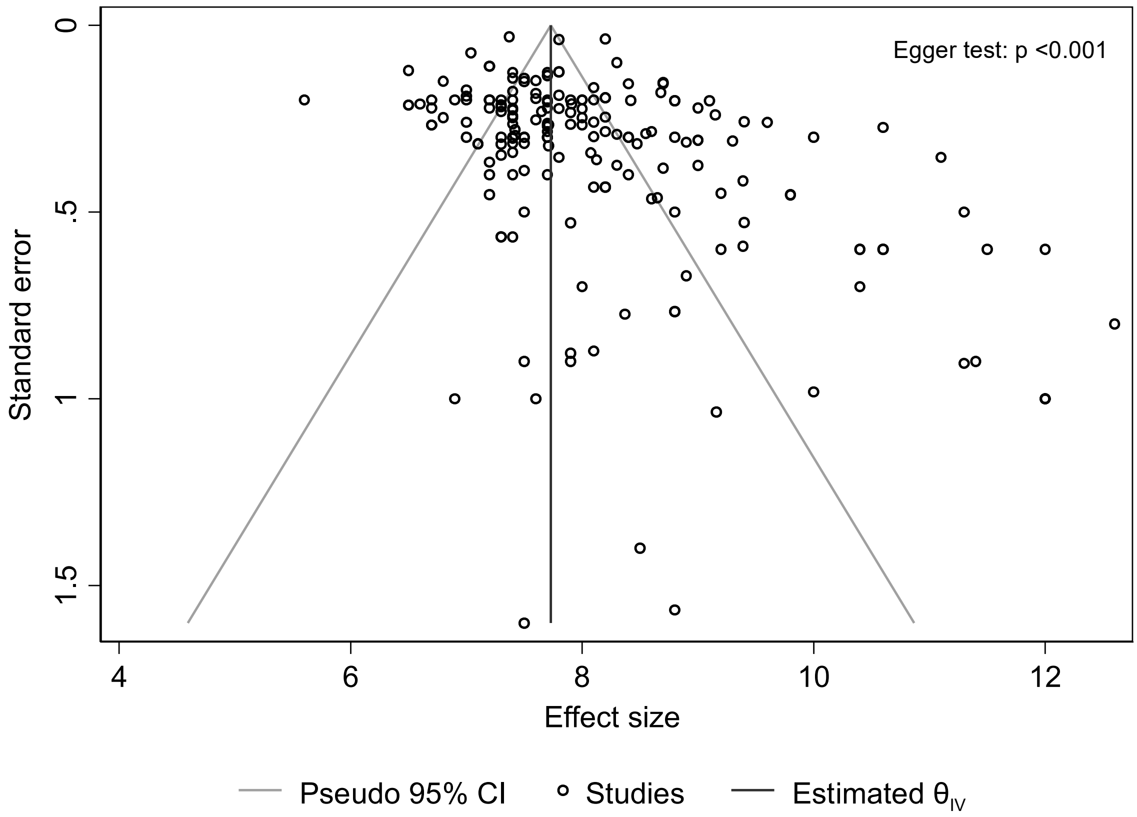

Table S5: Statistical analysis of HbA_{1c} of all included groups

```
. meta summarize, subgroup(age_grp2)
```

Study	Effect size	[95% conf.	interval]	% weight
Group: adolescents				
Dovc, K (a) 2017	7.500	7.205	7.795	0.61
Singhvi, A 2014	8.420	8.026	8.814	0.60
Francis, SL (a) 2015	8.600	8.042	9.158	0.58
Nguyen, T 2015 (a)	7.400	7.054	7.746	0.60
Matthys, D 1996	10.000	9.412	10.588	0.57
Poortmans, JR 1986 (a)	7.300	6.712	7.888	0.57
Komatsu, WR 2005	8.100	7.592	8.608	0.58
D'hooge, R (c) 2011	8.550	7.981	9.119	0.57
Faulkner, MS 2005	8.700	8.394	9.006	0.61
Adolfson, P 2012 (b)	8.100	7.773	8.427	0.60
Bjornstad, P 2018 (a)	9.000	8.396	9.604	0.57
Landt, KW 1985 (a)	12.000	10.040	13.960	0.32
Bjornstad, P 2015	8.500	5.756	11.244	0.22
D'hooge, R (a) 2011	8.125	7.419	8.831	0.55
Nadeau, KJ 2010	8.650	7.745	9.555	0.52
Bjornstad, P 2018 (d)	9.000	8.396	9.604	0.57
Adolfson, P 2012 (a)	7.600	7.103	8.097	0.58
Adolfson, P 2012 (c)	7.900	6.179	9.621	0.36
Poortmans, JR 1986 (b)	11.400	9.636	13.164	0.35
Bjornstad, P 2018 (e)	8.800	8.404	9.196	0.60
Francis, SL (c) 2015	8.400	8.093	8.707	0.61
Landt, KW 1985 (c)	12.000	10.040	13.960	0.32
Rowland, TW 1992	11.300	9.526	13.074	0.35
Kornhauser, C 2012	10.000	8.076	11.924	0.32
Abraham, MB 2017	7.800	7.107	8.493	0.55
Nguyen, T 2015 (b)	11.100	10.407	11.793	0.55
Gusso, S 2008	8.800	8.212	9.388	0.57
D'hooge, R (b) 2011	8.075	7.406	8.744	0.56
Roche, DM 2008 (a)	9.400	8.894	9.906	0.58
Roche, DM 2008 (b)	9.800	8.910	10.690	0.52
Michaliszyn, SF 2009	8.700	8.400	9.000	0.61
Dovc, K (b) 2017	7.900	7.443	8.357	0.59
Bjornstad, P 2018 (b)	8.800	8.404	9.196	0.60
Trigona, B 2010	8.200	8.128	8.272	0.62
D'hooge, R (d) 2011	8.475	7.853	9.097	0.57
Heyman, E 2007	8.100	7.515	8.685	0.57
Austin, A 1993	10.600	10.064	11.136	0.58
Roche, DM 2008 (c)	9.600	9.090	10.110	0.58
Landt, KW 1985 (b)	12.000	10.040	13.960	0.32
Landt, KW 1985 (d)	12.000	10.040	13.960	0.32
Bjornstad, P 2018 (c)	8.200	7.819	8.581	0.60
Bjornstad, P 2018 (f)	8.200	7.819	8.581	0.60
Gusso, S 2012	8.680	8.327	9.033	0.60
Giani, E 2018	7.400	6.925	7.875	0.59
Francis, SL (b) 2015	8.200	7.642	8.758	0.58
Dovc, K (c) 2017	7.700	7.437	7.963	0.61
**theta**	**8.802**	**8.480**	**9.123**	
Group: adolescents/adults				
Boff, W 2019 (f)	8.800	7.297	10.303	0.40
Lee, MJ 2016	7.900	7.380	8.420	0.58
McKewen, MW 1999	7.200	6.311	8.089	0.52
Faulkner, MS (a) 2010	9.400	8.365	10.435	0.49
Boff, W 2019 (e)	8.800	7.297	10.303	0.40
Jensen, T 1988 (a)	7.500	6.911	8.089	0.57
Jensen, T 1988 (c)	9.300	8.693	9.907	0.57
Guelfi, KJ 2007	7.700	7.177	8.223	0.58
Yardley, JE 2012	7.100	6.478	7.722	0.57
Zaharieva, DP 2016	7.400	6.965	7.835	0.59
Jensen, T 1988 (b)	8.700	7.950	9.450	0.54

Boff, W 2019 (c)	8.400	7.812	8.988	0.57
Yardley, JE 2013 (1)	7.100	6.478	7.722	0.57
Bussau, VA 2006	7.400	6.807	7.993	0.57
Boff, W 2019 (b)	8.200	7.351	9.049	0.53
Yardley, JE 2013 (2)	7.100	6.478	7.722	0.57
Shetty, VB 2018	8.000	7.515	8.485	0.59
Guelfi, KJ 2005	7.400	6.289	8.511	0.47
Boff, W 2019 (d)	8.400	7.812	8.988	0.57
Boff, W 2019 (a)	8.200	7.351	9.049	0.53
Faulkner, MS (b) 2010	9.390	8.230	10.550	0.46
Tagougui, S 2020	7.600	7.242	7.958	0.60
**theta**	**7.969**	**7.668**	**8.270**	
Group: adults				
Heise, T 2016	7.700	7.452	7.948	0.61
Bracken, RM 2012	9.160	7.130	11.190	0.31
Baldi, JC 2010	7.300	6.847	7.753	0.59
Atalay, M 1997	7.300	6.189	8.411	0.47
Brazeau, AS (1d) 2012	7.420	6.872	7.968	0.58
Tuominen, JA 1997	7.700	7.112	8.288	0.57
Rigla, M 2001 (b)	6.700	6.176	7.224	0.58
Campbell, MD (2) 2015	6.900	6.508	7.292	0.60
Rissanen, APE 2015	7.400	6.733	8.067	0.56
Wallberg-Henriksson, H 1986 (d)	10.600	9.424	11.776	0.46
West, DJ 2011 (a)	8.000	7.608	8.392	0.60
Roy-Fleming, A 2019	7.300	6.882	7.718	0.59
Rigla, M 2000 (a)	6.500	6.081	6.919	0.59
Bracken, RM 2011	8.300	8.104	8.496	0.61
Bally, L (2) 2016	7.000	6.628	7.372	0.60
Stettler, C 2005	7.400	6.915	7.885	0.59
Robitaille, M 2007	7.400	7.123	7.677	0.61
Laaksonen, DE 2000 (b)	8.000	7.562	8.438	0.59
Zaharieva, DP 2017	7.000	6.491	7.509	0.58
West, DJ 2011 (b)	8.000	7.608	8.392	0.60
Reddy, R 2012	7.400	6.780	8.020	0.57
Rigla, M 2001 (a)	6.500	6.081	6.919	0.59
Hilberg, T 2004	7.200	6.808	7.592	0.60
Veves, A 1997 (b)	9.800	8.911	10.689	0.52
Veves, A 1997 (c)	8.900	7.585	10.215	0.43
Tonoli, C 2015	7.040	6.895	7.185	0.62
Rissanen, APE 2018 (a)	7.300	6.676	7.924	0.57
Laaksonen, DE 1996	7.300	6.189	8.411	0.47
Bak, JF 1989	7.900	6.863	8.937	0.49
Franc, S 2015	7.900	6.136	9.664	0.35
Moser, O 2017	7.800	7.555	8.045	0.61
Ebeling, P (b) 1995	7.200	6.808	7.592	0.60
Wallberg-Henriksson, H 1982 (b)	11.300	10.320	12.280	0.50
Tagougui, S (2b) 2015	9.000	8.566	9.434	0.59
Rissanen, APE 2018 (b)	7.500	6.738	8.262	0.54
Peltonen, JE 2012	7.700	7.266	8.134	0.59
Roberts, TJ 2018 (2)	8.100	6.391	9.809	0.36
Komatsu, WR 2010 (b)	9.000	8.264	9.736	0.55
Lehmann, R 1997 (b)	7.500	5.736	9.264	0.35
Wilson, LC 2017	8.370	6.854	9.886	0.39
Peltoniemi, P 2001	7.000	6.412	7.588	0.57
Farinha, JB (d) 2018	8.000	7.477	8.523	0.58
Campbell, MD (1) 2015	8.100	7.708	8.492	0.60
Mccarthy, O 2020	6.800	6.506	7.094	0.61
Koponen, AS 2013	7.650	7.197	8.103	0.59
Bally, L (1) 2016	7.000	6.661	7.339	0.60
Brazeau, AS (2a) 2012	7.500	7.221	7.779	0.61
Brazeau, AS (1c) 2012	7.710	7.077	8.343	0.56
Campaigne, BN 1987	7.400	6.812	7.988	0.57
Gray, BJ 2016	9.200	8.024	10.376	0.46
Farinha, JB (f) 2018	7.200	6.766	7.634	0.59
Schneider, SH 1992 (b)	11.500	10.324	12.676	0.46
Fuchsjager-Mayrl, G 2002 (d)	7.000	6.608	7.392	0.60
Haagglund, H 2012	7.700	7.142	8.258	0.58
Benbassat, CA 2001	8.600	7.689	9.511	0.51

Heyman, E 2020	8.300	7.565	9.035	0.55
Murray, FT 1988	12.000	10.824	13.176	0.46
Ebeling, P (a) 1995	8.400	7.616	9.184	0.54
Schneider, SH 1992 (a)	12.600	11.032	14.168	0.38
Brazeau, AS (2c) 2012	7.600	7.310	7.890	0.61
Fuchsjager-Mayrl, G 2002 (c)	7.500	6.912	8.088	0.57
Raguso, CA 1995	8.000	6.628	9.372	0.42
Roberts, TJ 2018 (1)	7.700	7.297	8.103	0.60
Farinha, JB (b) 2018	7.200	6.481	7.919	0.55
Rigla, M 2000 (b)	6.700	6.176	7.224	0.58
Stewart, CJ 2017	7.400	7.152	7.648	0.61
Tagougui, S (1a) 2015	6.600	6.186	7.014	0.60
Valletta, JJ 2014	7.700	7.169	8.231	0.58
Laaksonen, DE 2000 (a)	8.200	7.718	8.682	0.59
Campbell, MD 2013	7.700	7.112	8.288	0.57
Moser, O 2019	7.200	6.808	7.592	0.60
Campbell, MD (2) 2014	6.700	6.266	7.134	0.59
Brazeau, AS (1a) 2012	7.710	7.188	8.232	0.58
Farinha, JB (e) 2018	7.500	6.880	8.120	0.57
Brazeau, AS (2b) 2012	7.700	7.184	8.216	0.58
Niranjan, V 1997 (a)	5.600	5.208	5.992	0.60
Brugnara, L 2012	6.900	4.940	8.860	0.32
Wallberg-Henriksson, H 1986 (b)	10.400	9.224	11.576	0.46
Farinha, JB (c) 2018	8.100	7.251	8.949	0.53
Fuchsjager-Mayrl, G 2002 (a)	7.300	6.908	7.692	0.60
Turinese, I 2017	7.370	7.310	7.430	0.62
Bussau, VA 2007	7.400	6.881	7.919	0.58
Wallberg-Henriksson, H 1982 (a)	10.400	9.028	11.772	0.42
Waclawovsky, G 2016	7.700	7.308	8.092	0.60
Wallberg-Henriksson, H 1986 (a)	10.400	9.224	11.576	0.46
Tagougui, S (1b) 2015	9.100	8.704	9.496	0.60
Farinha, JB (a) 2018	7.500	6.520	8.480	0.50
Chokkalingam, K 2007	7.900	7.508	8.292	0.60
Fuchsjager-Mayrl, G 2002 (e)	7.400	6.616	8.184	0.54
Niranjan, V 1997 (b)	8.800	7.820	9.780	0.50
Jenni, S 2008	6.700	6.308	7.092	0.60
Campbell, MD (1) 2014	7.700	6.916	8.484	0.54
Fuchsjager-Mayrl, G 2002 (f)	7.400	7.008	7.792	0.60
Goulding, R 2020	7.300	6.872	7.728	0.59
Lehmann, R 1997 (a)	7.600	5.640	9.560	0.32
Zebrowska, A 2018 (b)	7.200	6.985	7.415	0.61
Tuttle, KR 1988	8.800	5.732	11.868	0.19
Moser, O 2018 (2)	7.800	7.555	8.045	0.61
Sandoval, DA 2004	7.800	7.725	7.875	0.62
Zaharieva, DP 2019	6.500	6.262	6.738	0.61
Roberts, TJ 2020	7.800	7.363	8.237	0.59
Zebrowska, A 2018 (a)	7.200	6.985	7.415	0.61
Komatsu, WR 2010 (a)	7.500	4.362	10.638	0.18
Tagougui, S (2a) 2015	6.800	6.315	7.285	0.59
Wallberg-Henriksson, H 1986 (c)	10.600	9.424	11.776	0.46
Veves, A 1997 (a)	8.300	7.728	8.872	0.57
Fuchsjager-Mayrl, G 2002 (b)	7.700	7.112	8.288	0.57
Brazeau, AS (1b) 2012	7.420	6.843	7.997	0.57
Wanke, T 1992	9.200	8.318	10.082	0.52
Moser, O 2018 (1)	7.400	6.956	7.844	0.59
**theta**	**7.818**	**7.621**	**8.015**	
Group: other				
Fintini, D 2012	7.700	7.435	7.965	0.61
de Jesus, IC 2019	9.390	8.573	10.207	0.53
Seeger, JPH 2011	7.910	7.497	8.323	0.60
de Lima, VA 2017	9.150	8.680	9.620	0.59
Baraldi, E 1992	8.900	8.286	9.514	0.57
Al Khalifah, RA (b) 2016	7.600	7.215	7.985	0.60
Al Khalifah, RA (a) 2016	7.800	7.432	8.168	0.60
**theta**	**8.302**	**7.754**	**8.850**	
Overall				

theta	8.104	7.946	8.262

Heterogeneity summary

Group	df	Q	P > Q	tau2	% I2	H2
adolescents	45	469.91	0.000	1.058	96.19	26.25
adolescents/~s	21	83.00	0.000	0.373	78.06	4.56
adults	109	1143.17	0.000	0.965	96.83	31.58
other	6	52.68	0.000	0.484	91.42	11.65
Overall	184	2451.39	0.000	1.049	96.82	31.43

Test of group differences: Q_b = chi2(3) = 27.29   Prob > Q_b = 0.000
